# Adjuvant Hyperthermic Intravesical Chemotherapy in Intermediate- and High-Risk Non-muscle Invasive Bladder Cancer

**DOI:** 10.7759/cureus.45672

**Published:** 2023-09-21

**Authors:** Joana C Magalhães, Maria Sousa, Raquel Basto, Teresa Fraga, Inês Gomes, Catarina Fernandes, Mónica Mariano, Judy Paulo, Pedro Madeira, Gabriela Sousa

**Affiliations:** 1 Medical Oncology, Instituto Português de Oncologia de Coimbra Francisco Gentil, E.P.E., Coimbra, PRT; 2 Medical Oncology, Centro Hospitalar Universitário do Porto, Porto, PRT; 3 Medical Oncology, Centro Hospitalar Vila Nova de Gaia/Espinho, Gaia, PRT; 4 Medical Oncology, Centro Hospitalar Universitário de São João, Porto, PRT; 5 Medical Oncology, Centro Hospitalar e Universitário de Coimbra, Coimbra, PRT

**Keywords:** adjuvant intravesical chemotherapy, hyperthermic intravesical chemotherapy, mitomycin c, bacillus calmette-guérin, non-muscle invasive bladder cancer

## Abstract

Introduction: Non-muscle invasive bladder cancer (NMIBC) is a frequently diagnosed neoplasm, which is typically managed with transurethral resection of bladder tumor (TURBT) eventually followed by intravesical therapies. Bacillus Calmette-Guérin (BCG) is used as first-line adjuvant treatment in high- (HR) and intermediate-risk (IR) NMIBC, although, in the latter, mitomycin C (MMC) may also be used. Multiple limitations to the use of BCG encouraged the search for therapeutic alternatives. In this context, hyperthermic intravesical chemotherapy with MMC (HIVEC-MMC) emerged as a promising therapy in the adjuvant setting for NMIBC. The aim of our study was to evaluate the tolerability, compliance, and survival outcomes of HIVEC-MMC in patients with IR- and HR-NMIBC.

Material and Methods: This was a single-center retrospective analysis of IR- and HR- NMIBC patients who received HIVEC-MMC after TURBT between August 2018 and August 2022. Levels of risk stratification were defined using the European Association of Urology (EAU) criteria. The protocol consisted of four weekly HIVEC-MMC instillations (induction) followed by six monthly instillations (maintenance). The primary outcomes were to evaluate the tolerability and compliance with the HIVEC-MMC protocol and secondary outcomes were disease-free survival (DFS) and overall survival (OS). For the purpose of statistical analysis, methods of descriptive statistics, survival analysis (Kaplan-Meier estimation), and multivariate analysis (Cox regression, and binary logistic regression) were used.

Results: Fifty-seven patients were enrolled with a median age of 67.9 (34.4-83.5) years old. In this cohort, 40 patients (70.2%) had primary tumors. At the time of referral for HIVEC-MMC, the majority of the patients had IR-NMIBC (n= 33, 57.9%). A total of 41 patients (71.9%) completed the HIVEC-MMC protocol. Disease recurrence and adverse events (AEs) were the most common reasons to stop the protocol. After a median follow-up of 31 months (95% CI, 5.0-54.0), 32 patients (61.4%) were disease-free, 22 (38.6%) experienced recurrent disease and six patients (10.5%) died, although only one death was directly attributable to bladder cancer. The median DFS was 42 months (95% CI, 28.0-56.0). Completion of the HIVEC-MMC maintenance phase protocol stood as a predictive factor for DFS (44 months, 95% CI 29.1-58.9 vs. 14 months, 95% CI 0.0-29.6, p < 0.001; HR 4.48, 95% CI 1.65-12.15). The median OS was not reached; the 24- and 48-month OS were 92.6% and 82.7%, respectively. EAU risk group, ECOG-PS, and completion of HIVEC protocol were found to be significant predictive factors of OS but lost their significance on multivariate analysis. However, if we exclude those who experienced recurrence during the maintenance phase protocol, treatment completion had a significant positive impact on OS (HR: 42.8, 95% CI 1.75-1045.072, p= 0.021).

Conclusions: Our study suggests that HIVEC is a secure and well-tolerated treatment with promising efficacy data, making this therapeutic approach a feasible option in IR- and HR-NMIBC patients, mainly in those who cannot tolerate or have contraindications to BCG therapy, but also as an alternative during BCG shortages.

## Introduction

Bladder cancer is the most common malignancy of the urinary system [1], and, in Portugal, it represents the seventh-most frequently diagnosed tumor [2]. Approximately 75% of the patients present with non-muscle invasive bladder cancer (NMIBC) [3]. As a result of variable recurrence and progression rates depending on risk stratification, long-term surveillance is mandatory and patients frequently require multiple intravesical and surgical procedures, resulting in a significant financial burden associated with the management of this particular cancer [4,5].

NMIBC is typically managed with transurethral resection of bladder tumor (TURBT), eventually followed by intravesical therapies; bacillus Calmette-Guérin (BCG) is used as first-line adjuvant treatment in high- (HR) and intermediate-risk (IR) NMIBC. However, in the latter case, mitomycin C (MMC) may also be used [3,6]. Limitations to BCG (higher toxicity risk than MMC and BCG shortages due to limited supply) in addition to the fact that a considerably high percentage of patients experience recurrence and progression to a muscle-invasive disease (MIBC) despite adequate treatment encouraged the search for therapeutic alternatives [7-9].

The main focus of these treatments has been to develop device-assisted technology to improve the delivery of already established oncological agents, most commonly MMC, a cytotoxic antibiotic that leads to cell death by DNA alkylation and cross-linking of DNA [10,11]. In this context, hyperthermic intravesical chemotherapy (HIVEC) allows the administration of MMC at a temperature of 43°C (± 0.5ºC). Increased temperature not only has a direct impact on cancer cells (by altering intracellular metabolism and causing DNA damage) but also increases the solubility of the pharmacological agent and improves the permeability of cell membranes, favoring drug penetration. In this process, heat shock proteins are released from cancer cells, activating dendritic cells, T cells, and NK cells, further enhancing its antineoplastic effect [12].

The present study aimed to retrospectively evaluate tolerability, compliance, and efficacy outcomes of HIVEC-MMC in patients with IR - and HR-NMIBC.

## Materials and methods

Study design

This was a non-interventional, retrospective, single-center study approved by the Institutional Ethics Committee. In our institution, the data of NMIBC patients treated with adjuvant intent with HIVEC-MMC using de COMBined Antineoplasic Thermotherapy Bladder Recirculation System (COMBAT BRS®, Combat Medical, Wheathampstead, UK) with MMC between August 2018 and August 2022 were analyzed. MMC (40 mg) was diluted in distilled water (50 mL), heated at 43°C (± 0.5ºC) outside the body, and then recirculated through a closed system via a three-way catheter at a stable pressure and constant temperature and flow rate for 60 minutes using this device. The HIVEC-MMC protocol consisted of four weekly instillations (induction) followed by six monthly instillations (maintenance). Response to treatment was assessed with urinary cytology and cystoscopy performed four weeks after the last HIVEC instillation and then repeated every three or six months (upon the categorization in HR or IR, respectively), or when clinically indicated. Toxicity was scored according to Common Toxicity Criteria for Adverse Events version 5 (CTCAE).

Study population and subgroups

Clinical data from all patients who were treated with HIVEC-MMC between August 2018 and August 2022 were reviewed. Eligible patients were required to have the following: (1) histological diagnosis of NMIBC; (2) IR- and HR-NMIBC according to the European Association of Urology (EAU) criteria for risk stratification; (3) age of at least 18 years; (4) Eastern Cooperative Oncology Group performance status (ECOG-PS) grade 0-1.

History of prior or concomitant carcinoma in situ (CIS), T2 bladder cancer, urothelial carcinoma in the upper urinary tract at the time of the diagnosis, treatment with chemotherapy or pelvic radiotherapy during the last six months, and documented allergic reaction to MMC in the past were considered exclusion criteria.

Study assessments and outcomes

Information was obtained from the electronic clinical records of included patients. Data regarding demographics, ECOG-PS, tumor characteristics, current and past treatment details, response evaluation data, and survival rates were collected.

We evaluated the tolerability and compliance with the HIVEC-MMC protocol. Adverse events (AE) were graded according to the CTCAE.

Regarding the efficacy outcomes, we evaluated disease-free survival (DFS) and overall survival (OS). DFS was defined as the time from TURBT to the earliest date of identification of recurrent disease (including disease progression) or death from any cause. Recurrence was defined as the emergence of an NMIBC (pTa, pT1). Progression was defined as tumor stage pT ≥ 2 at TURBT. OS was defined as the time from TURBT to the occurrence of death from any cause.

Statistical analysis

Categorical variables were described using frequency and percentages, and continuous variables using median and range. Survival analysis was performed using the Kaplan-Meier estimate and Cox regression for multivariate analysis. Exploratory analysis was performed using the chi-square test, Fisher's exact test, and binary logistic regression for categorical data; the Mann-Whitney U test was used for continuous data. Statistical significance was defined as a p-value < 0.05. Data analysis was performed using IBM SPSS Statistics for Windows®, Version 27.0 (IBM Corp., Armonk, NY).

## Results

Baseline characteristics

A total of 57 subjects were enrolled in the study, 33 (57.9%) in the IR-NMIBC subgroup and 24 (42.1%) in the HR-NMIBC. A summary of the baseline characteristics of the participants is presented in Table 1. The median age at diagnosis was 67.9 years old (95% CI 34.4 - 83.5) and 44 patients (77.2%) were male. All patients had an ECOG-PS score of 0-1.

**Table 1:** Baseline characteristics of patients who received HIVEC-MMC BCG: Bacillus Calmette-Guérin; BMI: body mass index; IR-NMIBC: intermediate-risk non-muscle invasive bladder cancer; HR-NMIBC: high-risk non-muscle invasive bladder cancer; MMC: mitomycin-C

Variables	Overall population (n=57)	IR-NMIBC (n=33)	HR-NMIBC (n=24)
Gender – n (%)			
Male	44 (77.2)	26 (78.8)	18 (75.0)
Female	13 (22.8)	7 (21.2)	6 (25)
Age (years old)			
Median (range)	67.9 (34.4 - 83.5)	61.5 (34.4 - 81.1)	75.3 (45.2 – 83.5)
BMI (kg/m^2^)			
Median (range)	28.4 (19.7 – 44.2)	27.8 (19.7 - 39.8)	28.4 (21.5 - 44.2)
Smoking Status – n (%)			
Never	22 (38.6)	12 (36.4)	10 (41.7)
Past Smoker	29 (50.9)	15 (45.5)	14 (58.3)
Current Smoker	6 (10.5)	6 (18.2)	-
Diagnosis Type – n (%)			
New Diagnosis	40 (70.2)	19 (57.6)	21 (87.5)
Recurrence	17 (29.8)	14 (42.4)	3 (12.5)
Previous Treatment (if recurrent tumors)			
MMC	3 (5.3)	3 (9.1)	-
BCG	1 (1.8)	-	1 (4.2)
Tumour Stage – n (%)			
pTa	39 (68.4)	32 (97.0)	7 (29.2)
pT1	18 (31.6)	1 (3.0)	17 (70.8)

In this cohort, 40 patients (70.2%) had a primary cancer diagnosis, whereas the remaining 17 (29.8%) presented with a recurrent tumor and had already undergone previous treatments; none of the patients had been treated with device-assisted intravesical therapies before, however, three (5.3%) had received intravesical chemotherapy with normothermic MMC and one (1.8%) intravesical immunotherapy with BCG.

At the time of referral for HIVEC-MMC, 39 patients (68.4%) had pTa tumors and 18 (31.6%) had pT1; regarding the grade, 49 (86%) were grade 2, and 8 (14%) were grade 3.

Treatment compliance and tolerability

A total of 41 patients (71.9%) completed the HIVEC-MMC protocol; 50 patients (87.7%) completed the induction and 43 (75.4%) the maintenance phase. The median number of instillations received was 10 (SD: 2.4). Data regarding the compliance and tolerability of the planned treatment are summarized in Table 2. Disease recurrence and AEs were the most common reasons to stop protocol, however, three patients (5.3%) decided to withdraw consent because of logistical problems, namely the time expenditure associated with repeated hospital visits.

**Table 2:** HIVEC-MMC protocol compliance and treatment tolerability IR-NMIBC: intermediate-risk non-muscle invasive bladder cancer; HIVEC-MMC: hyperthermic intravesical chemotherapy with mitomycin-C; HR-NMIBC: high-risk non-muscle invasive bladder cancer

Variables	Overall Population (n=57)	IR-NMIBC (n=33)	HR-NMIBC (n=24)
Completion of HIVEC-MMC treatment (%)			
Yes	41 (71.9)	25 (75.8)	16 (66.7)
No	16 (28.1)	8 (24.2)	8 (33.3)
Completion of induction – n (%)			
Yes	50 (87.7)	30 (90.9)	20 (83.3)
No	7 (12.3)	3 (9.1)	4 (16.7)
Completion of maintenance – n (%)			
Yes	43 (75.4)	27 (81.8)	16 (66.7)
No	14 (24.6)	6 (18.2)	8 (33.3)
Reasons for not completing the treatment – n (%)			
Recurrence	6 (10.5)	3 (9.1)	3 (12.5)
Urethral Stricture	2 (3.5)	2 (6.1)	-
Adverse Events	7 (12.3)	3 (9.1)	4 (16.7)
Patient Choice	3 (5.3)	-	3 (12.5)
Death	1 (1.8)	-	1 (4.2)
Adverse events – n (%)			
Yes	24 (42.1)	15 (45.5)	9 (37.5)
No	33 (57.9)	18 (54.5)	15 (62.5)

Overall, 33 patients (57.9%) did not experience any adverse events (AEs), whereas 24 (42.1%) experienced at least one AE of any grade throughout the treatment duration. The total number of registered AEs was 39. Their severity was evaluated as grade 1-2; none of the patients experienced grade ≥3 AEs. Table 3 shows the distribution and severity of AEs. The most common treatment-related AE was dysuria (36.8%).

**Table 3:** Adverse events related to HIVEC-MMC HIVEC-MMC: hyperthermic intravesical chemotherapy with mitomycin-C

Adverse Events Description – n (%)	Grade 1 – n (%)	Grade 2 – n (%)	Total – n (%)
Dysuria	21 (36.8)	-	21 (36.8)
Suprapubic pain	4 (7.0)	4 (7.0)	8 (14.0)
Urinary tract infection	-	5 (8.8)	5 (8.8)
Hematuria	-	4 (7.0)	4 (7.0)
Urinary retention	1 (1.8)	-	1 (1.8)

Efficacy outcomes

After a median follow-up of 31 months (95% CI, 5.0-54.0), 32 patients (61.4%) were disease-free, and 22 (38.6%) experienced recurrent disease; of these, 20 patients (35.1%) had local recurrence and two (3.5%) progressed to MIBC. The median time until local recurrence and disease progression was 18.0 months (95% CI, 0.3-43.0) and 26 months (95% CI, 14.0-37.0), respectively. Six patients (10.5%) died, but only one death (1.8%) was directly attributable to bladder cancer.

**Figure 1:**
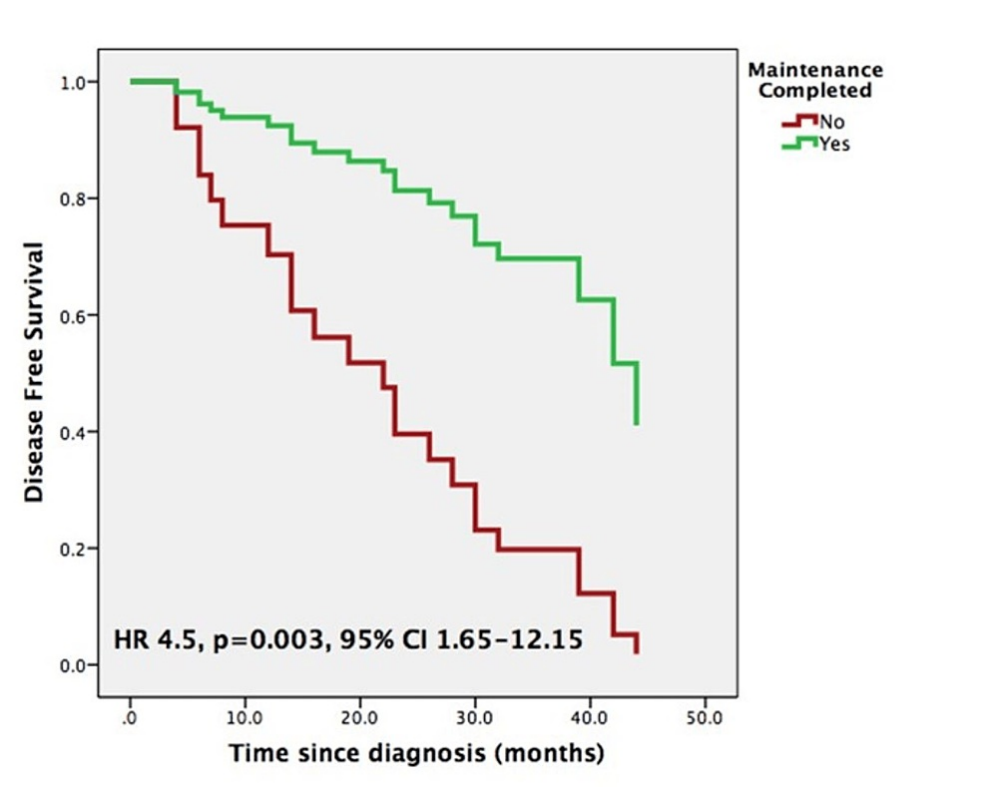
Multivariate analysis (Cox regression) for disease-free survival – maintenance completed factor

The median DFS was 42 months (95% CI, 28.0-56.0). The 24- and 48-month DFS rates were 72.1% and 33.4%, respectively. The IR-NMIBC group exhibited a higher median DFS (44 months, 95% CI 27.0-61.0) than the HR-NMIBC group (39 months, 95% CI 22.0-56.0), although this difference did not reach statistical significance (p=0.740). The exploratory analysis also showed that completion of the HIVEC-MMC protocol stood as a predictive factor for DFS: patients who completed HIVEC-MMC exhibited a median DFS that was 16 months longer compared to those who did not (44 months, 95% CI 27.0-62.0 vs. 28 months, 95% CI 1.0-55.0, p<0.001). However, on multivariate analysis, only the completion of the maintenance phase protocol remained statistically significant (HR 4.5, p=0.003, 95% CI 1.65-12.15) (Figure 1).

After recurrence or progression, five patients (8.8%) underwent active surveillance, seven (12.3%) were treated with intravesical BCG instillations, one (1.8) with normothermic intravesical MMC, five (8.8%) were rechallenged with HIVEC-MMC, two received systemic chemotherapy (3.5%), one (1.8%) underwent radical cystectomy and one (1.8%) was recommended best supportive care.

**Figure 2:**
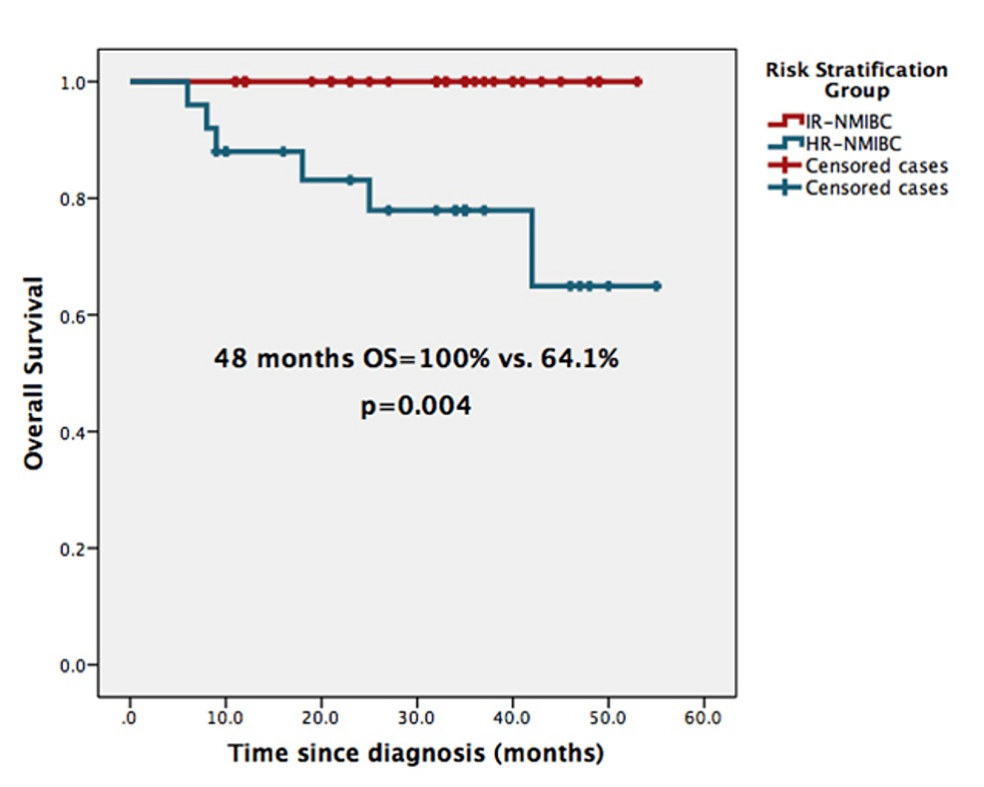
Overall survival according to risk stratification group

Median OS was not reached; the 24- and 48-month OS were 92.6% and 82.7%, respectively. IR-NMIBC group (vs. HR-) and completion of HIVEC-MMC protocol were found to be significant predictive factors of better OS. The 48-month-OS rate for patients with IR-NMIBC was 100% and for patients with HR-NMIBC was 64.1% (p=0.03) (Figure 2). For patients who completed HIVEC-MMC, the 48-month-OS rate was 96.6%, and 49.2% for those who did not complete the protocol (p=0.001). In the multivariate analysis, when we excluded patients who experienced recurrence before finishing HIVEC-MMC, completion of the maintenance phase protocol sustained a positive impact on OS (HR: 42.8, 95% CI 1.75-1045.072, p= 0.021).

## Discussion

In this study, we present an initial analysis of the real-world data regarding the use of adjuvant HIVEC-MMC in patients diagnosed with IR- and HR-NMIBC in a single Portuguese institution. The relevance of our results is reinforced by the current context, where there is a growing interest in seeking alternatives to BCG treatment, including devices to enhance the efficacy of chemotherapeutic agents.

It is important to highlight that the HIVEC-MMC protocol implemented at our institution differs from the majority of the protocols adopted by other institutions. Our treatment regimen comprises an induction phase consisting of four weekly instillations, as described by Sousa et al. [10], but, frequently, six weekly instillations are used [13-16]. This difference has the potential to positively influence tolerability outcomes, but also to compromise the efficacy ones, since existing data suggest that longer treatment duration is an independent prognostic factor, particularly in primary tumors and IR-NMIBC [17].

Our primary objective was to assess tolerability and compliance with the HIVEC-MMC protocol. Overall, HIVEC-MMC was a well-tolerated adjuvant treatment and showed a favorable toxicity profile. Our results are in alignment with literature showing that reported AEs during treatment are relatively common, but usually are low grade (CTCAE grade 1 or 2) and self-limited [13,15,17,18]. The most frequent AE in this cohort was dysuria, followed by suprapubic pain and urinary tract infections. It is worth noting that lower urinary tract symptoms may not result exclusively from the toxic effect of HIVEC-MMC but may also be related to the multiple intravesical procedures that these patients undergo.

Concerning treatment compliance, the induction phase was well-tolerated (87.7% completion) and a total of 71.9% of patients completed the whole protocol. The most common reasons for not completing HIVEC-MMC were intolerability and recurrence, but it is important to point out that three patients (5.3%), discontinued treatment because they considered that the burden associated with time expenditure related to travels to the hospital and HIVEC-MMC instillations did not outweigh the treatment benefits. The age of these patients and the fact that their treatment took place during the COVID-19 pandemic could be reasons that explain their decisions, but this travel and time “toxicity” had already been described by Sylvester et al. and Conroy et al. [4,13].

We also aimed to explore clinical outcomes. Given that our study is a retrospective analysis and lacks a comparative arm, drawing conclusions regarding efficacy becomes challenging. Nonetheless, our 24-month-DFS was 72.1%, which is markedly higher than the data reported by Tan et al. in the HIVEC II trial [19]; this difference is particularly noteworthy when considering that, contrary to HIVEC II, our study population comprised 42.1% of individuals with HR-NMIBC. The fact that median DFS time was significantly higher in patients who completed the HIVEC-MMC protocol allows us to infer the good results obtained with this therapeutic and reinforces that, as described by Plata et. al, treatment duration is a prognostic factor in preventing tumor recurrence and mortality [17].

The 24-month-OS was 92.6%, which is similar to the data described in other studies [16,17]. In our population, death was observed in six patients, but since only one death was directly attributable to bladder cancer, this may more likely reflect patients’ frailty characteristics (such as advanced age and comorbidities), than the consequences of the tumor itself.

However, this study has some limitations: this was a non-randomized retrospective study in a single institution, which included a small and heterogeneous cohort, with a short follow-up time; our results should be interpreted with caution since retrospective comparisons are prone to multiple confounding factors. Despite these limitations, real-world data is extremely important to externally validate clinical trials’ results. Large-scale prospective and randomized studies are needed to determine the optimal MMC dosage and schedule for HIVEC-MMC (potentially including a long-term maintenance regimen), as well as to evaluate long-term safety and efficacy outcomes.

## Conclusions

To our knowledge, this is the inaugural study divulging safety and efficacy outcomes derived from the utilization of adjuvant HIVEC-MMC in IR- and HR-NMIBC within the specific context of a Portuguese population. HIVEC-MMC has demonstrated itself as a secure and well-tolerated treatment and efficacy data also proved to be promising. The reported findings resonate with the established body of evidence and suggest that HIVEC-MMC might be considered a viable option in IR- and HR-NMIBC patients who cannot tolerate or have contraindications to BCG therapy, but also as an alternative during BCG shortages.
